# Corticosterone induces neurotoxicity in PC12 cells via disrupting autophagy flux mediated by AMPK/mTOR signaling

**DOI:** 10.1111/cns.13212

**Published:** 2019-08-18

**Authors:** Run‐Dong Ma, Gui‐Juan Zhou, Miao Qu, Ji‐Hong Yi, Ya‐Ling Tang, Xiang‐Yi Yang, Ya‐Xiong Nie, Hong‐Feng Gu

**Affiliations:** ^1^ Department of Neurology of the First Affiliated Hospital University of South China Hengyang China; ^2^ Department of Physiology & Institute of Neuroscience University of South China Hengyang China; ^3^ Institute of Neuroscience of the First Affiliated Hospital University of South China Hengyang China

**Keywords:** AMPK, autophagy, corticosterone, mTOR, neurotoxicity

## Abstract

**Aims:**

Our previous study indicated that chronic stress caused autophagy impairment and subsequent neuron apoptosis in hippocampus. However, the mechanism underlying the stress‐induced damage to neurons is unclear. In present work, we investigated whether stress‐level glucocorticoids (GCs) GCs promoted PC12 cell damage via AMPK/mTOR signaling‐mediated autophagy.

**Methods:**

Chronic stress‐induced PC12 cell injury model was built by treatment with high level corticosterone (CORT). Cell injury was evaluated by flow cytometry assay and transmission electron microscopy observation.

**Results:**

Autophagy flux was measured based on the changes in LC3‐II and P62 protein expressions, and the color alteration of mCherry‐GFP‐LC3‐II transfection. Our results showed that CORT not only increased cell injury and apoptosis, but also dysregulated AMPK/mTOR signaling‐mediated autophagy flux, as indicated by the upregulated expression of LC3‐II and P62 proteins, and the lowered ration of autolysosomes to autophagosomes. Mechanistically, our results demonstrated that autophagy activation by AMPK activator metformin or mTOR inhibitor rapamycin obviously promotes cell survival and autophagy flux, improved mitochondrial ultrastructure, and reduced expression of Cyt‐C and caspase‐3 in CORT‐induced PC12 cells.

**Conclusion:**

These results indicate that high CORT triggers PC12 cell damage through disrupting AMPK/mTOR‐mediated autophagy flux. Targeting this signaling may be a promising approach to protect against high CORT and chronic stress‐induced neuronal impairment.

## 1 INTRODUCTION

Accumulated evidences have confirmed that elevated glucocorticoids (GCs), resulting from chronic stress and prolonged or excessive use of GCs, can induce neurotoxicity and cognitive dysfunction.^1^, ^2^, ^3^, ^4^ However, the underlying mechanisms for GCs‐triggered these damaging effects have not been fully elucidated. To clarify the detrimental influence of high concentration of GCs on neuronal cells, increasing attention has been given to hippocampal neuron pathology.^5^, ^6^ It has been shown that stress‐level of corticosterone (CORT), a major glucocorticoid, results in pathological damage to neurons in hippocampus.^7^ Although our previous study indicated that chronic unpredictable mild stress (CUMS) significantly increased CORT level and neuron cell lost in the hippocampus CA1 region and contributed to cognition impairment of rats, the underlying mechanism by which stress‐induced high GCs level exerts neurotoxicity on hippocampal neurons is still largely unknown.^8^

Autophagy is an essential pathway for cell survival via degrading the dysfunctional cellular components and the damaged organelles. Autophagy flux, a dynamic process of autophagy, is featured by formatting autophagosomes (APs), fusing APs with lysosomes to form autolysosomes (ALs), and degrading the cargoes sequestered in ALs.^9^, ^10^ Thus, disrupted autophagy flux can result in aggregation of the damaged organelles, and thereby contributing to cell injury and death. Impaired autophagy flux is closely correlated with pathogenesis of neurodegenerative diseases.^11^, ^12^ In recent years, several studies have shown that abnormal autophagy is responsible for GCs‐induced spinal cord and SH‐SY5Y cell injury.^13^, ^14^ Our previous study found that CUMS promotes neuron apoptosis of hippocampal CA1 region via suppressing autophagy, but the relationship between stress‐induced high GCs level and autophagy flux dysfunction in neuron cells has not been identified.^8^ Therefore, further elucidating the mechanisms for these phenomena is beneficial to preventing neurotoxicity induced by high concentration of GCs.

AMP‐activated protein kinase (AMPK), a upstream signaling molecule of rapamycin complex 1 (mTORC1), plays a critical role in regulating various cellular processes such as energy metabolism and autophagy.^15^, ^16^, ^17^ The activation of AMPK depends on phosphorylation of its threonine 172.^15^ Its activation facilitates autophagy through inhibiting mTORC1 activity. Several studies have indicated that excess glucocorticoids exposure significantly altered AMPK activity in a tissue‐dependent manner.^18^, ^19^, ^20^ Furthermore, inactivation of AMPK has been revealed to be associated with CORT‐induced neurotoxicity.^21^ Collectively, these reports suggest that AMPK/mTOR signaling‐mediated autophagy may be involved in GCs‐induced damage to neurons.

Based on the above data, we speculated that high GCs would dysregulate AMPK/mTOR signaling in PC12 cells, thus contributing to autophagy flux impairment and cell death. To test this hypothesis, PC12 cells were treated with CORT to establish stress cell model. First, we explored the influences of CORT on cell injury, AMPK/mTOR signaling, and autophagy flux. Then, AMPK activator Met and mTOR inhibitor RAP were used to confirm whether CORT‐induced PC12 cell injury via disrupting AMPK/mTOR signaling‐mediated autophagy flux. Our results indicate that excess CORT promotes PC12 cell damage by impairing autophagy flux via inactivating AMPK and activating mTOR.

## 2 MATERIALS AND METHODS

### 2.1 Materials

Rat pheochromocytoma PC12 cell line was purchased from Cell Bank of Shanghai Institute of life Science (Chinese Academy of Sciences). Corticosterone, rapamycin (RAP), and metformin (Met) were obtained from Sigma‐Aldrich. Primary antibodies to AMPK, phosphor‐AMPK (T172), phosphor‐mTOR (S2448), GAPDH were purchased from Cell Signaling Technology. Primary antibodies to LC3‐I/II, p62, Cytochrome c (Cyt‐c), caspase‐3 were from Abcam; Annexin V Apoptosis Detection Kit was supplied by eBioscience. Fetal bovine serum (FBS) and Dulbecco's modified Eagle's medium (DEME) were from Gibco BRL.

### 2.2 Cell culture

PC12 cells were cultured in 25 cm^2^ flasks containing DMEM supplemented with 10% FBS, 100 U/mL penicillin, and 100 μg/mL streptomycin. Then, cells were incubated in a humidified atmosphere containing 5% CO_2_ at 37°C. For each experiment, PC12 cells in logarithmic phase were used.

### 2.3 Cell viability assay

Cell viability of PC12 cells was performed by MTT method. The cells were seeded in 96‐well plates at a density of 1 × 10^4^ cells per well containing 10% FBS medium. After 24 hours incubation, the medium was replaced with FBS‐free DMEM. Then, the cells were incubated with CORT at various concentrations 0.1, 1, and 10 μmol/L. After treatment with indicated agents for 24 hours, the cells were incubated with MTT solution (0.5 mg/mL, 30 μL per well) for additional 4 hours in the dark. Then, 100 μL DMSO was added to each well and the absorbance value was measured by spectrophotometer at 570 nm.

### 2.4 Annexin V‐FITC/PI double staining flow cytometry

Cell apoptosis was evaluated by flow cytometry with annexin V‐FITC/PI double staining.^22^ Briefly, PC12 cells were seeded in 6‐well plates at density of 1 × 10^6^ cells/well. After incubated with indicated agents for 24 hours, the cells were digested with trypsin‐EDTA and collected in Eppendorf tubes by centrifugation. The harvested cells were resuspended in 400 μL PBS at density of 10 × 10^5^ cells/mL. Then, cell apoptosis analysis was performed by flow cytometry with annexin V‐FITC/PI double staining. Cell apoptosis rate of PC12 cells was calculated by counting the number of apoptotic cell per 1 × 10^4^ cells utilizing flow cytometry (FCM; BD Bioscience).

### 2.5 Western blotting

Western blot analysis was carried out as previously described.^22^ Briefly, after treating with indicated agents for 24 hours, PC12 cells were collected and washed three times with cold PBS. All the cells were lysed in ice‐cold RIPA buffer (Cell Signaling Technology) with protease inhibitor/protein phosphatase inhibitors, and total protein was extracted by centrifugation. Protein contents were assessed by a bicinchoninic acid protein assay kit. Equal amounts of the denatured proteins (10‐20 µg per lane) were separated by a 10% sodium dodecyl sulfate polyacrylamide gel electrophoresis. Subsequently, the proteins were transferred to a polyvinylidene difluoride membrane (Millipore) membrane and blocked in a blocking buffer containing 5% BSA for 2 hours. The membranes were then incubated at 4°C overnight with primary antibodies against, respectively, LC3‐I/II (1:1000), P62 (1:1000), p‐AMPK (1:1000), p‐mTOR (1:1000), Cyt‐c (1:1000), cleaved caspase‐3 (1:1000), and GAPDH (1:2000). After washing three times with TBST (TBS containing 0.1% Tween 20), the membranes were incubated 2 hours with secondary antibody conjugated to horseradish peroxidase (1:5000). Finally, the bands were detected by an enhanced chemiluminescence system (ECL, Millipore). The intensity of each bolt was quantified with Image J software (National Institutes of Health) and normalized to that of GAPDH.

### 2.6 Adenovirus (Ad)‐mCherry‐GFP‐LC3‐II transfection and confocal microscopy

PC12 cells were cultured in 24‐well plates in a density of 5 × 10^4^ cells/well and transfected with adenovirus expressing mCherry‐GFP‐LC3‐II plasmid at a viral titer of 30 multiplicities of infection for 24 hours. Subsequently, the cells were treated with various agents at indicated concentration for another 24 hours. Then, the images of PC12 cells transfected with Ad‐GFP‐RFP‐LC3‐II were visualized with a Leica TCS SP5 laser scanning confocal microscopy. Autophagy flux was measured by the color change of mCherry‐GFP. At least 30 cells of each group were analysed.

### 2.7 Transmission electron microscopy

The ultrastructural observation including mitochondrial morphometry and APs/ALs were performed by transmission electron microscopy (TEM) as previously described.^22^, ^23^ In brief, PC12 cells were treated with indicated agents for 24 hours. After removing culture medium and washing with PBS for three times, the cells were fixed with 2.5% glutaraldehyde for 1 hour at 4°C. After postfixation in 1% OsO4 for another 1h, the cells were dehydrated and embedded in Araldite. Ultra‐thin sectioning (40‐60 nm) was performed and the sections were double‐stained with uranyl acetate and lead citrate. The sections were then observed under a JEOL 1230 TEM.

### 2.8 Statistical analysis

All the values are presented as the mean ± SEM and analysed by GraphPad Prism 7.0 (GraphPad Software, Inc). The statistical significance was determined by one‐way analysis of variance (ANOVA) followed by a post hoc Dunnett's test to compare the means of individual groups. The difference was considered significant when *P* < .05.

## 3 RESULTS

### 3.1 High concentration of CORT reduces the activity of PC12 cells

**Figure 1:**
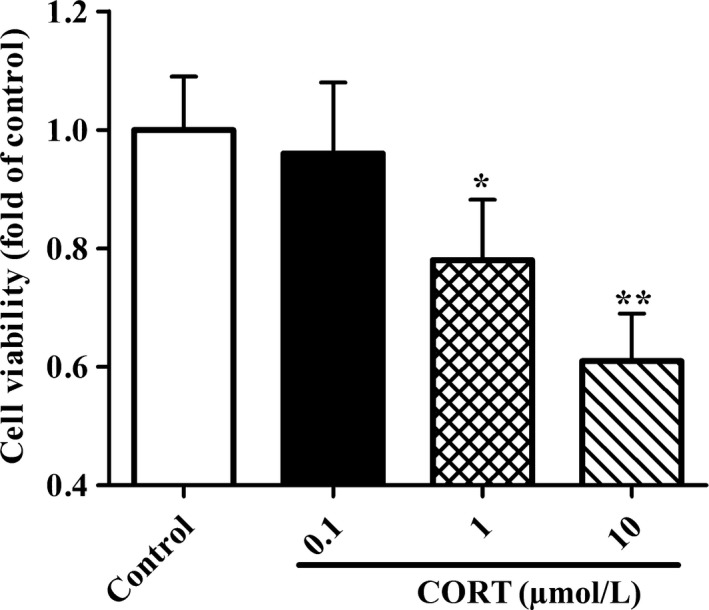
Effects of CORT on cell viability of PC12 cells. PC12 cells were incubated with CORT at different concentrations (0.1, 1, and 10 μmol/L) for 24 h. Then, PC12 Cell viability was measured by MTT assay kit. All the data were presented as mean ± SEM of three dependent experiments. *P < .05, **P < .01 vs control group

To confirm the cytotoxic effect of CORT on neuronal cells, PC12 cells were treated with CORT at a concentration of 0.1, 1, and 10 μmol/L for 24 hours, respectively. Then, cell survival rate was measured by MTT assay. As shown in Figure 1, there was no significant difference in cell viability between 0.1 μmol/L group and the control group, indicating that the physiological level of CORT had no significant damage to the cells. As expected, with the increase of CORT concentrations, the PC12 cell viability decreased dramatically. These results manifested that the stress‐like levels of CORT significantly declined the activity of PC12 cells in a dose‐dependent manner with the maximal influence at 10 μmol/L concentration.

### 3.2 High concentration of CORT increases apoptosis rates of PC12 cells

**Figure 2:**
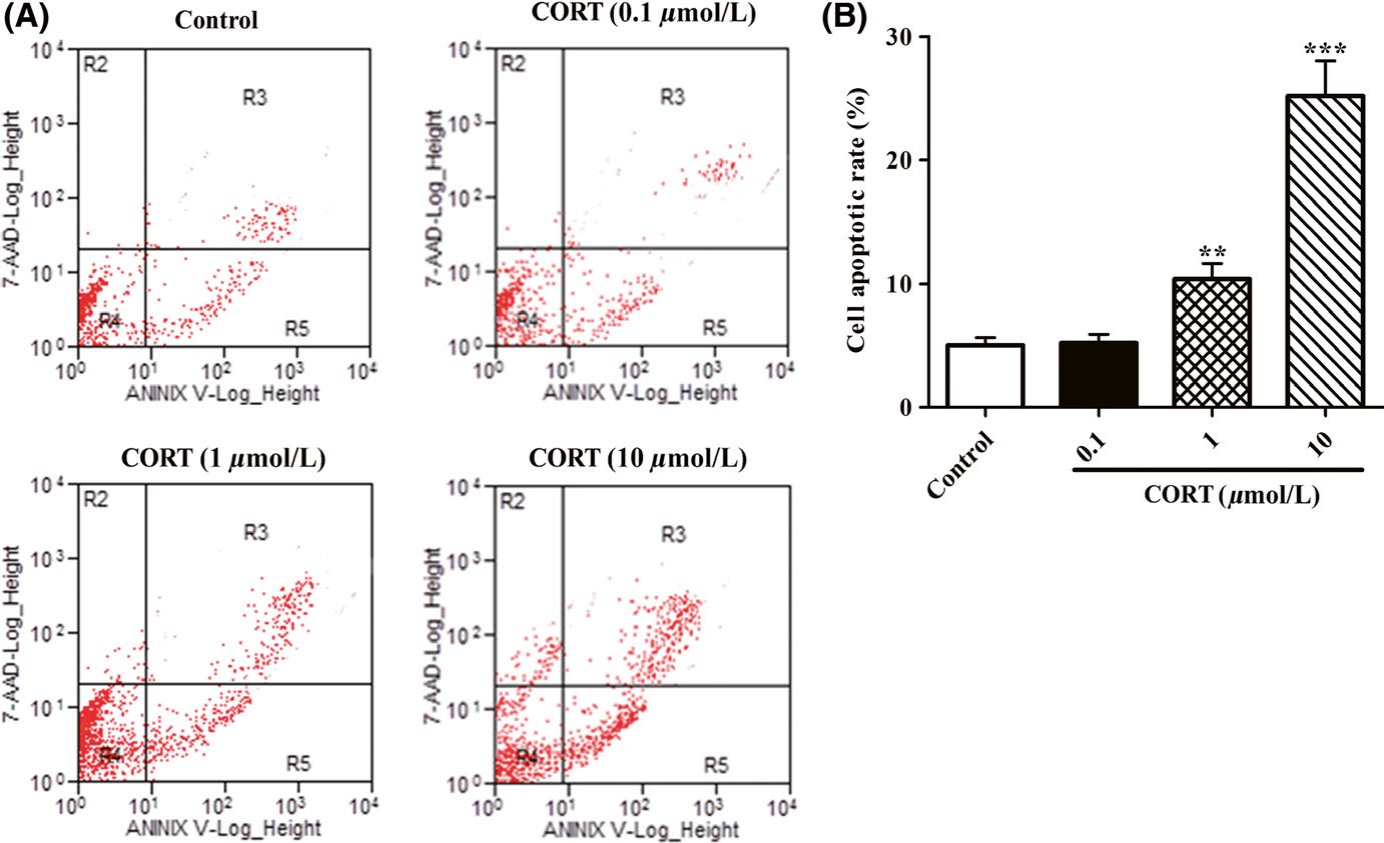
Effect of CORT on cell apoptosis of PC12 cells. PC12 cells were incubated with indicated concentrations of CORT (0.1, 1, and 10 μmol/L) in 6‐well plates for 24 h. Then, cell apoptotic rates of PC12 cells were evaluated by flow cytometry with annexin V/PI double staining as described in Section 2. A, Representative images of cell apoptotic rates analysed by flow cytometry with annexin V/PI double staining. B, The percentage of PC12 cell apoptosis was quantified as described in Section 2. Data were presented as mean ± SEM of three dependent experiments. **P < .01, ***P < .001 vs control group

To test the effects of CORT on PC12 cell apoptotic death, cell apoptosis rates were detected by flow cytometry. As shown in Figure 2A,B, there was no significant difference in the apoptosis rate between control group and 0.1 μmol/L CORT group. However, the apoptotic rates of PC12 cells were significantly elevated both in 1 μmol/L CORT group and 10 μmol/L CORT group as compared with those in the control group. The results indicate that physiological concentration CORT has no significant influence on apoptosis of PC12 cells, while high concentration of CORT profoundly promotes PC12 cell apoptosis in a dose‐dependent manner.

### 3.3 High levels of CORT dysregulates AMPK/mTOR pathway and impairs autophagy flux in PC12 cells

**Figure 3:**
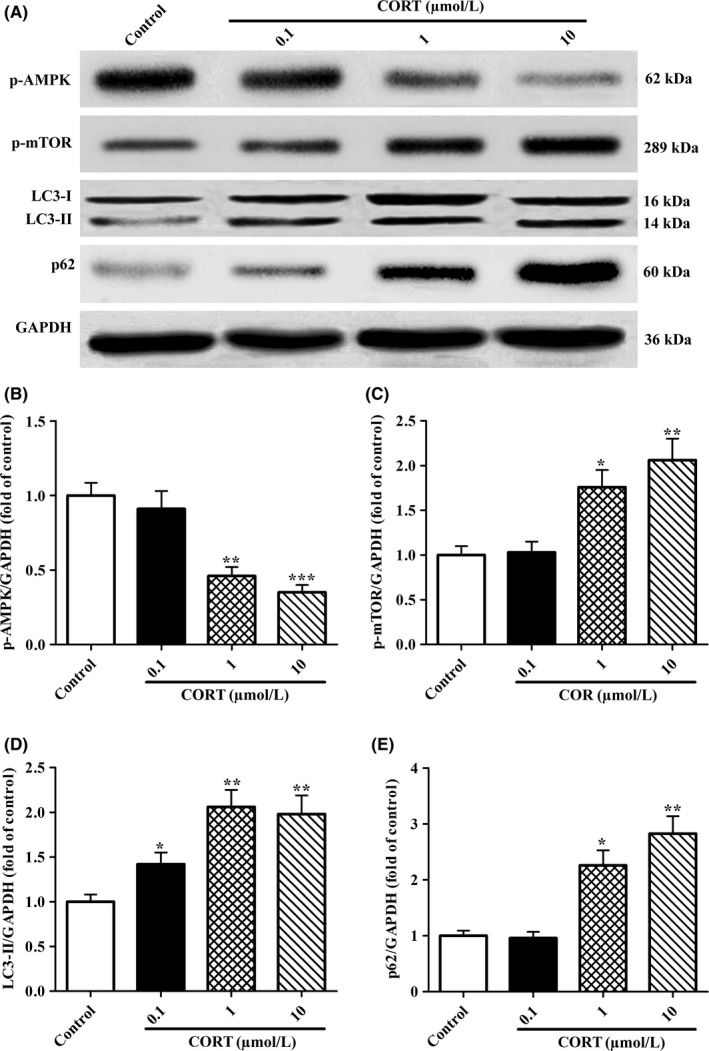
Effects of CORT on the expression of p‐AMPK, p‐mTOR, LC3‐II, P62 proteins in PC12 cells. PC12 cells were administrated with various concentrations of CORT (0.1, 1, and 10 μmol/L) for 24 h. A, Western blot analysis was performed to measure the protein levels of p‐AMPK, p‐mTOR, LC3‐I (16 kDa) and LC3‐II (14 kDa), and p62 (60 kDa) in PC12 cells. Each lane was loaded with 10 µg proteins in all experiments. B‐E, p‐AMPK, p‐mTOR, LC3‐II, and p62 levels were analysed with Sigma Scan Pro5 software. Values are the mean ± SEM (n = 3). *P < .05, **P < .01, ***P < .001 vs control group

Since AMPK/mTOR signaling plays a critical role in neuronal cell death and survival,^15^, ^24^ we hypothesized that this signaling may be implicated in neurotoxicity to PC12 cells induced by high concentrations of CORT. The expressions of phosphorylated AMPK (p‐AMPK) and mTOR (p‐mTOR) in PC12 cells treated with different doses of CORT were measured by Western blotting assay. As shown in Figure 3A,B, CORT significantly lowered the expressions of p‐AMPK in a dose‐dependent manner with the maximal influence at 10 μmol/L concentration. In contrast, the levels of p‐mTOR (Figure 3A,C) were elevated in PC12 cells treated with CORT as compared with the control ones. These findings indicate that high concentration of CORT diminishes AMPK phosphorylation and enhances mTOR phosphorylation in PC12 cells.

Considering AMPK phosphorylation takes an important role in regulation of autophagy via negatively regulates mTOR activation, we then asked whether high levels of CORT influenced autophagy flux in PC12 cells. In present study, two classic autophagy‐associated markers, LC3‐II and p62 in PC12 cells, were determined by Western blotting analysis. As depicted in Figure 3A,D,E, CORT exposure caused an increase both in expressions of LC3‐II and P62 with a dose‐dependent manner. Given that P62 protein is degraded with LC3‐II‐formed APs and is inversely proportional to autophagy flux, the elevated LC3‐II protein levels in PC12 cells exposed to CORT were resulted from autophagy flux blocking. Taken together, these results imply that stress‐like levels CORT impair PC12 cell autophagy flux and cell viability, with a maximal impairment at 10 μmol/L. Therefore, 10 μmol/L CORT was selected for the subsequent experiments.

### 3.4 High level of CORT disrupts autophagy flux via dysregulating AMPK/mTOR pathway in PC12 cells

**Figure 4:**
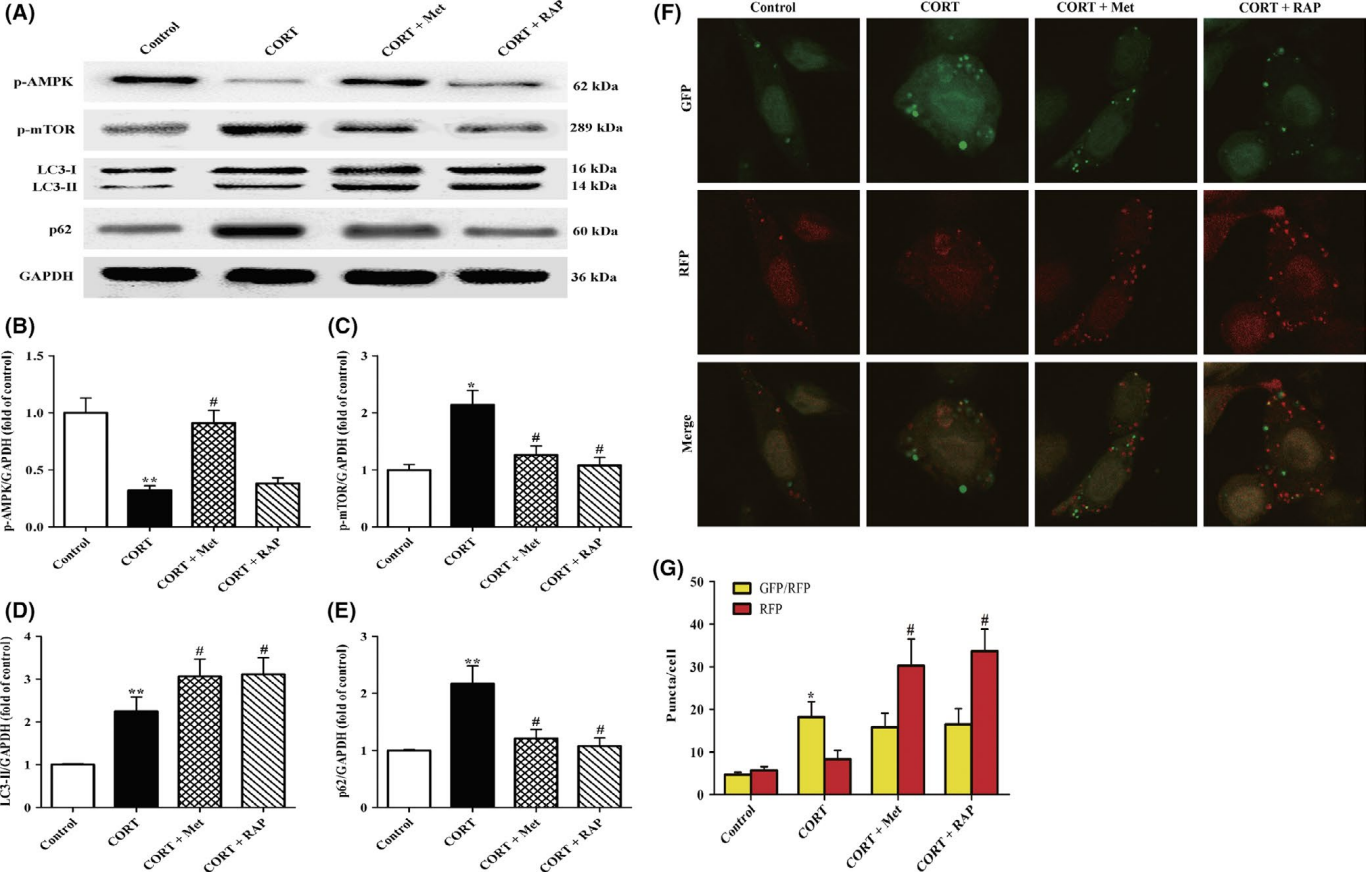
Effects of treatment with Met or RAP on AMPK/mTOR‐mediated autophagy flux in PC12 cells induced by high CORT. A, PC12 cells were incubated with CORT (10 μmol/L) in the presence of Met (2 mmol/L) or RAP (200 nmol/L). After co‐treatment for 24 h, all the cells were harvested and proteins were extracted from cell lysates. Western blot was then carried out to determine the protein levels of GAPDH (36 kDa), p‐AMPK, p‐mTOR, LC3‐I (16 kDa) and LC3‐II (14 kDa), and p62 (60 kDa) in PC12 cells. Each lane was loaded with 10 μg of proteins for all experiments. B‐E, The relative optical density values of p‐AMPK, p‐mTOR, LC3‐II, and p62 to GAPDH were analysed by Sigma Scan Pro5 software, respectively. F, Representative confocal images of GFP and RFP fluorescent puncta in PC12 cells. Cells were transfected with Ad‐mCherry‐GFP‐LC3‐II plasmid for 24 h. After treatment with indicated reagents for another 24 h, RFP (red color)‐GFP (green color)‐LC3‐II puncta were observed under laser scanning confocal microscopy. G, Quantification of GFP/RFP double‐positive and RFP single‐positive dots in each cell (n = 32 cells/group). Data were presented as mean ± SEM. *P < .05, **P < .01 vs control group; ^#^ *P* < .05 vs CORT group

To identify whether AMPK/mTOR pathway is responsible for the impaired autophagy flux induced by high levels of CORT, PC12 cells were incubated with Met (an AMPK activator) and RAP (a mTOR inhibitor), respectively. Then, the expressions of p‐AMPK, p‐mTOR, LC3‐I, LC3‐II, and p62 protein were evaluated by Western blot analysis. As shown in Figure 4A‐C, the decreased p‐AMPK and increased p‐mTOR expression in CORT + PC12 cells were remarkably reversed by treatment with Met and RAP, respectively. Accordingly, Met and RAP treatments significantly enhanced the expression of LC3‐II (Figure 4A,D) and decreased the expression of P62 (Figure 4A,E) in CORT + PC12 cells. These results reveal that activation of AMPK or inhibition of mTOR reverses autophagy flux impairment induced by CORT in PC12 cells.

To further confirm whether CORT impaired autophagy flux in PC12 cells via AMPK/mTOR signaling, the cells were transfected with the tandem monomeric GFP‐RFP‐LC‐3‐tagged protein and the changes of APs and ALs were analysed by confocal microscopy. Given that RFP fluorescence persists even in acidic condition of the lysosome lumen where GFP loses its fluorescence, yellow puncta (the colocalization of GFP and RFP fluorescence) in merged images represented APs, whereas the solely red ones indicated ALs. The changes in the number of red and yellow puncta represent altered autophagy flux. As shown in Figure 4F,G, yellow and green dots (indicating APs) in CORT group were significantly increased without a corresponding increase in red dots (indicating ALs) compared with those in the control group, confirming an impairment of autophagy flux induced by CORT in PC12 cells. As expected, although yellow puncta were not augmented both in CORT + Met group and CORT + RAP group, red dots were notably increased as compared with those in the CORT group. These results indicate that activation of AMPK or inhibition of mTOR can rescue the impaired autophagy flux induced by CORT in PC12 cells.

### 3.5 CORT promotes the ultrastructure damage via impairing AMPK/mTOR‐mediated autophagy in PC12 cells

**Figure 5:**
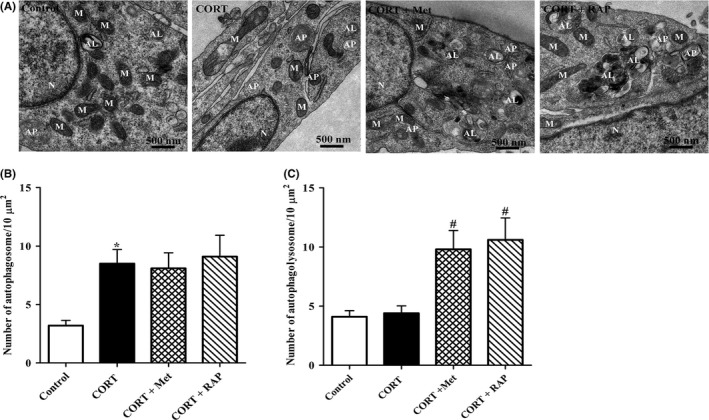
Effects of treatment with Met or RAP on the ultrastructures of PC12 cells induced by high CORT. A, Representative TEM images of PC12 cells. PC12 cells were incubated with CORT (10 μmol/L) in the presence of Met (2 mmol/L) or RAP (200 nmol/L) for 24 h. Cells were then harvested for TEM observation as described in Section 2. Mitochondria (M), nucleus (N), autophagosome (AP), and autolysosomes (ALs) were indicated. B, C, The number of APs and ALs was quantified (n = 12 cells/group). All the data were presented as mean ± SEM. *P < .05 vs control group; ^#^ *P* < .05 vs CORT group. Scale bar, 500 nm

To identity whether CORT exerted its neurotoxicity through AMPK/mTOR‐mediated autophagy flux defect, PC12 cells were treated with CORT together with Met or RAP for 24 hours, and then, the morphological ultrastructural alterations were observed under the TEM. As shown in Figure 5A,B, the number of APs significantly increased, while the number of ALs increased slightly in CORT group when compared with those in the control group. Consistent with the impaired autophagy flux, those cells in CORT group manifested the characteristics of damaged cells, featured by chromatin condensation, mitochondria swelling and the mitochondrial cristae fragmented or disappeared when compared with the normal structure of the controls. However, co‐treatment with Met not only reversed the impaired autophagy flux, indicated by a slight decrease in the number of APs and a significantly increase in ALs, but also obviously improved the ultrastructural characters of PC12 cells when compared with CORT group. The same tendency was observed in CORT + RAP group. Collectively, these results further confirm that AMPK/mTOR‐mediated autophagy flux disruption is responsible for detrimental effects of high CORT level on PC12 cells.

### 3.6 Restoring AMPK/mTOR pathway‐mediated autophagy protects PC12 cells against cell apoptosis induced high CORT

**Figure 6:**
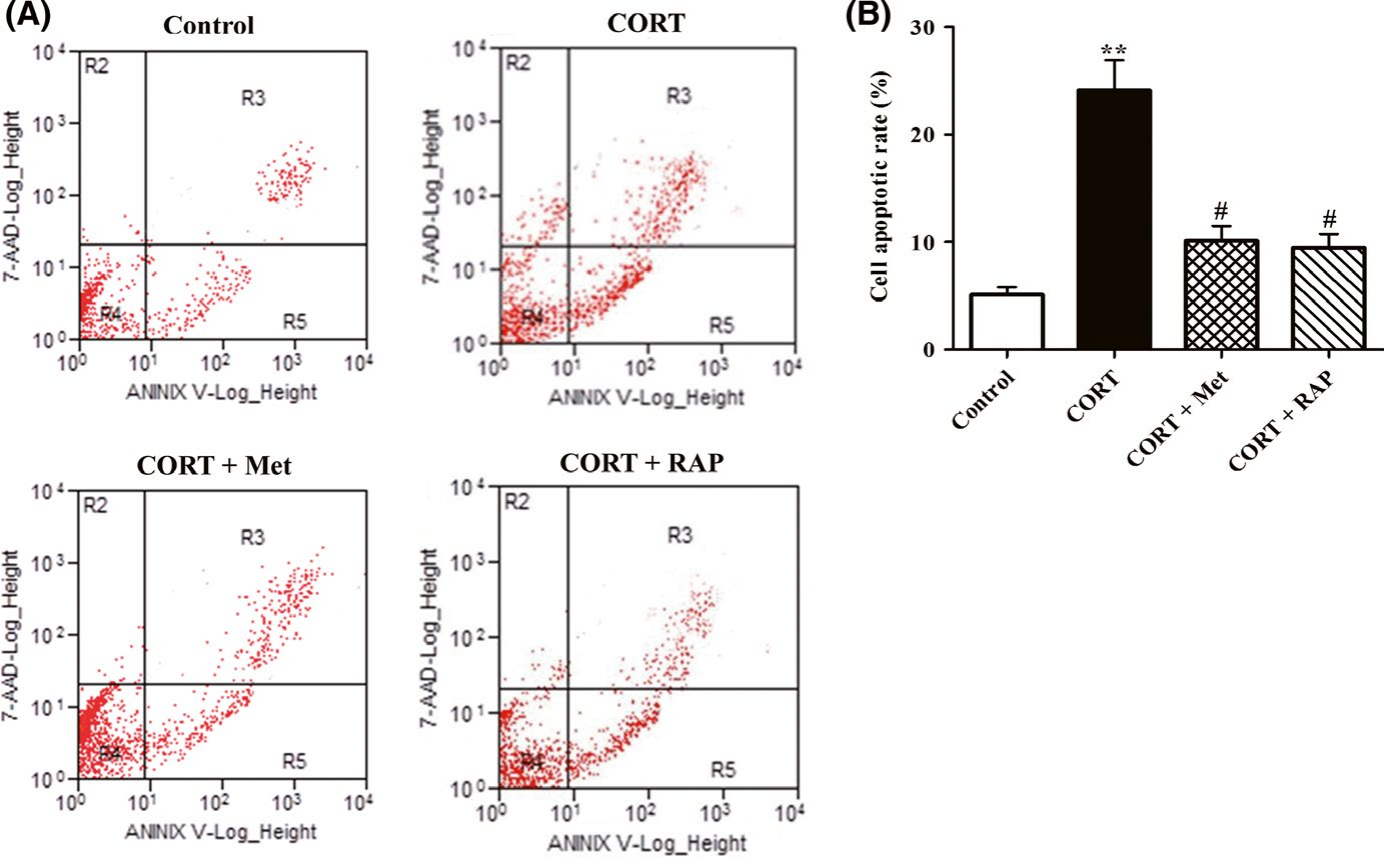
Effects of treatment with Met or RAP on PC12 cell apoptosis induced by high CORT. PC12 cells were exposed to CORT (10 μmol/L) in the presence of Met (2 mmol/L) or RAP (200 nmol/L). After treatment for 24 h, cells were harvested, and then, cell apoptotic rates of PC12 cells were measured by flow cytometry. A, Representative images of cell apoptotic rates determined by flow cytometry with annexin V/PI double staining. B, Quantitative analyses the percentage of PC12 cell apoptosis. Data were presented as mean ± SEM. ^#^ *P* < .05 vs CORT group, **P < 0.01vs control group

To further clarify inhibition of autophagy by CORT contributing to neurotoxicity on PC12 cells, we investigated whether activation of autophagy by Met or RAP could reverse cell apoptosis in PC12 cells exposed to CORT. In present study, cell apoptotic rate of each group was analysed by flow cytometry. As shown in Figure 6A,B, cell apoptotic rates of CORT group were significantly increased as compared with those of the control group. Interestingly, the cell apoptotic rates both in Met + CORT group and RAP + CROT group were significantly declined as compared with those in CORT group, indicating that activation of autophagy by co‐treatment with Met or RAP profoundly protected PC12 cells against CORT‐induced cell apoptosis. Collectively, these results clearly showed that activation of autophagy mediated by AMPK/mTOR signaling obviously abrogated CORT neurotoxicity on PC12 cells.

### 3.7 Restoring AMPK/mTOR pathway‐mediated autophagy reduces the release of Cyt‐c and caspase‐3 induced by CORT in PC12 cells

**Figure 7:**
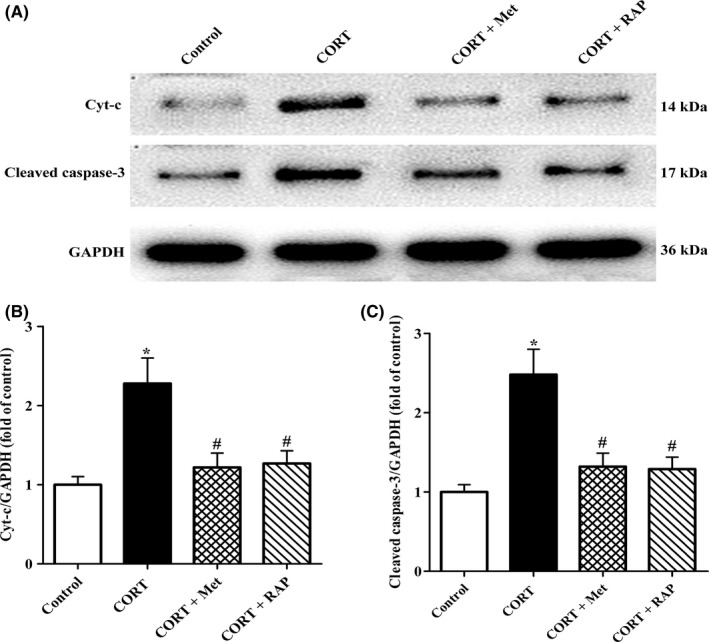
Effects of treatment with Met or RAP on proapoptotic protein Cyt‐c and cleaved caspase‐3 expression in PC12 cells exposed to high CORT. PC12 cells were administrated with CORT (10 μmol/L) in the presence of Met (2 mmol/L) or RAP (200 nmol/L) for 24 h. All the cells were collected, and then, proteins were extracted from cell lysates for Western blot analysis. Each lane was loaded with 20 μg of proteins for all experiments. A, Representative blots of GAPDH (36 kDa), Cyt‐c (14 kDa), and cleaved caspase‐3 (17 kDa) in PC12 cells. B, C, Quantitative analyses of the relative optical density values of Cyt‐c and cleaved caspase‐3 to GAPDH were performed by Sigma Scan Pro5 software. All the data were expressed as mean ± SEM. *P < .05 vs control group, *^#^P* < .05 vs CORT group

Considered that Cyt‐c released from the damaged mitochondria exerts a critical role in cell apoptosis via activating caspase‐3,^25^ the expressions of Cyt‐c and cleaved caspase‐3 in PC12 cells were determined by Western blot assay. As shown in Figure 7A‐C, the levels of Cyt‐c and cleaved caspase‐3 proteins in CORT group were elevated dramatically as compared with those of the control group. As expected, the expressions of Cyt‐c and cleaved caspase‐3 were significantly lowered both in CORT + Met group and CORT + RAP group when compared with those in CORT group. These results confirm that activation of autophagy by Met or RAP inhibits Cyt‐c release and the cleaved caspase‐3 production, thereby rescuing CORT‐induced PC12 cell apoptotic death.

## 4 DISCUSSION

In the present study, we investigated the influences of stress‐level GCs on PC12 cell injuries and the underlying mechanism. We found that high level CORT exposure decreased cell viability and increased cell apoptosis. Furthermore, CORT treatment significantly suppressed the phosphorylation of AMPK and enhanced the phosphorylation of mTOR. It was also manifested that CORT exposure impaired autophagy flux in PC12 cells. Notably, AMPK activation by Met or mTOR inhibition by RAP restored autophagy flux and attenuated CORT‐induced neurotoxicity in PC12 cells. These findings indicate that autophagy flux dysfunction mediated by AMPK/mTOR signaling in the present of stress‐level CORT contributes to the reduced cell viability and increased cell apoptotic rate in PC12 cells.

The PC12 cells administrated with high level of CORT to induce the neuronal injury have been used as an in vitro experimental model of cognitive dysfunction.^21^, ^26^, ^27^, ^28^ In present work, we established a cell model of stress in PC12 cells by treatment with different concentration of CORT, and confirmed that CORT notably decreased PC12 cell vitality and exacerbated cell death in a dose‐dependent manner. Furthermore, the morphological ultrastructural alterations in PC12 cells also indicated the neurotoxicity of CORT. Consistently, stress‐level CORT could exert obvious neurotoxicity on the cultured primary hippocampal neurons as well as PC12 cells. To clarify the mechanism by which stress‐level CORT‐induced PC12 cells damage, we explored the AMPK/mTOR in which CORT involved, including changes in phosphorylation of AMPK and mTOR, autophagy flux impairment, mitochondrial damage, Cyt‐C release, and caspase‐3 activation.

It has been known that AMPK/mTOR signaling mediates cell survival and plays a critical role in regulating autophagy.^15^, ^17^, ^28^ AMPK, a metabolic sensor, which favors neuronal survival through guarding mitochondrial homeostasis and energy balance. Several studies have implied that high CORT promotes neuronal cell death via inhibiting the activation of AMPK.^21^, ^29^ In present study, our results revealed a key role of this signaling in CORT‐induced PC12 cell damage. 10 µmol/L CORT significantly lowered AMPK phosphorylation and enhanced mTOR phosphorylation, and increased cell apoptosis of PC12 cells. Met is a major therapeutic agent for treating patients with type 2 diabetes. The ability of Met to treat this common metabolic disorder requires activation of AMPK signals, but its exact molecular mechanism is still a mystery.^30^ In this work, Met treatment not only reversed the influences of CORT on AMPK activation, but also attenuated PC12 cell death and mitochondrial structure damage resulting from CORT. Same neuroprotective effects were observed in PC12 cells treatment with mTORC1 inhibitor RAP. These results demonstrate that high CORT‐induced neurotoxicity to PC12 cell is via dysregulating AMPK/mTOR signaling pathway.

Autophagy is essential for cell survival via removing the damaged/senescence organelles and degenerating abnormal folded proteins.^31^, ^32^ It has been confirmed that activation of AMPK inhibits mTOR and induces autophagy in neurons.^33^ Therefore, we further evaluated the alterations of autophagic flux in PC12 cells for determining whether AMPK/mTOR‐mediated autophagy impairment accounted for CORT‐induced neurotoxicity. Changes in expression of LC3‐II and p62 protein are often used to measure autophagic flux. The results of Western blotting analysis indicated that CORT markedly increased LC3‐II and p62 expression. Since the intracellular protein levels of p62 are negatively correlated with autophagy flux,^34^, ^35^ the results implied that autophagy flux was impaired in PC12 cells incubated with CORT. Moreover, dual fluorescence mRFP‐GFP‐LC3‐II plasmids transfection and TEM assay results further confirmed that CORT severely disrupted autophagy flux in PC12 cells. Of note, both Met and RAP treatment abolished the autophagic flux impairment induced by CORT on PC12 cells. Our findings are consistent with GY Yang's study that autophagy activation by glycyrrhizic acid alleviates CORT‐induced neurotoxicity in SH‐SY5Y cells.^14^ These results indicate that CORT impaired autophagy flux of PC12 cells through dysregulating AMPK/mTOR pathway.

Mitochondria not only regulates cell metabolism and energy balance, but also control cell death.^36^, ^37^ Damaged mitochondrial results in proapoptotic protein Bax translocating from the cytoplasm to its outer membrane, causing mitochondrial membrane potential decline. Subsequently, Cyt‐C is released from mitochondria into the cytosol.^38^ The released Cyt‐C activates caspase‐3 in an Apaf‐1 and caspase 9‐dependent manner.^39^ In present work, our TEM results indicated that CORT‐treated PC12 cells exhibited obvious mitochondrial ultrastructure damage, including mitochondria swelling and the mitochondrial cristae fragmented. Consistent with TEM results, Western blot analysis dada revealed that CORT exposure significantly enhanced Cyt‐C release and caspase‐3 activation, implying that CROT‐induced PC12 cell apoptosis via mitochondrial apoptotic pathway. Interesting, the above proapoptotic effects of CORT on PC12 cells were canceled by Met and RAP treatment, respectively.

In conclusion, CORT‐induced neurotoxicity on PC12 cells is a possible mechanism for hippocampal neuron injuries under chronic stress. This neurotoxic effects of CORT are via disrupting AMPK/mTOR‐mediated autophagy flux, which results in aggregation of the damaged mitochondrial and activation of mitochondria‐mediated apoptotic pathway. Regulation of AMPK/mTOR signaling to restore neuronal cell autophagy flux may represent a potential novel therapeutic approach for CORT and chronic stress‐induced neurodegenerative diseases.

## CONFLICT OF INTEREST

The authors declare no conflict of interest.
